# Interleukins in diagnosis of perinatal asphyxia: A systematic review

**DOI:** 10.18502/ijrm.v17i5.4598

**Published:** 2019-06-26

**Authors:** Hassan Boskabadi, Ali Moradi, Maryam Zakerihamidi

**Affiliations:** ^1^ Department of Pediatrics, Faculty of Medicine, Mashhad University of Medical Sciences, Mashhad, Iran.; ^2^ Orthopedic Research Center, Mashhad University of Medical Sciences, Mashhad, Iran.; ^3^ Department of Midwifery, Faculty of Medical Sciences, Islamic Azad University, Tonekabon Branch, Tonekabon, Iran.

**Keywords:** Diagnosis, Interleukins, Asphyxia neonatorum.

## Abstract

**Background:**

Biochemical markers including interleukins (ILs) has been proposed for early diagnosis of asphyxia

**Objective:**

This study has aimed to systematically review the significance of IL measurements in the diagnosis of perinatal asphyxia.

**Materials and Methods:**

PubMed, Cochrane Library, Web of Science, Embase, and Scopus databases before 2017 were searched for the following keywords: asphyxia, neonatal, interleukin, and diagnosis. A total of 13 out of 300 searched papers were finally selected for evaluation. Interleukins under study were IL6 and interleukin 1β (IL-1β). Interleukins had been measured in 10 studies by serum samples, 2 studies by samples of Cerebro Spinal Fluid (CSF), and 1 study by sample of umbilical cord blood. The inclusion criteria were: studies on neonates, with adequate information from the test results and studies using markers other than ILs to detect asphyxia; however, studies with only abstracts available were excluded.

**Results:**

Research on the issue suggests that IL6 > 41 Pg/dl has the sensitivity of 84.88% and the specificity of 85.43%, whereas IL-1β > 4.7 Pg/dl has the sensitivity of 78% and specificity of 83% in the diagnosis of neonatal asphyxia. Among diagnostic ILs for neonatal asphyxia, combination of IL6 and IL-1β had the highest sensitivity, that is, 92.9%.

**Conclusion:**

IL6 and IL-1β of serum samples were used in the early diagnosis of perinatal asphyxia and are useful predictors for the outcomes of perinatal asphyxia and its intensity. In addition, simultaneous evaluation of IL-1β and IL6 can improve the sensitivity of the early diagnosis of perinatal asphyxia.

## 1. Introduction

Perinatal asphyxia has been defined as the lack of oxygen that occurs either before, during, or after birth (1). It is a significant cause of perinatal morbidity and mortality as well as neurological disabilities in the surviving babies (2, 3). Annually, four million babies are born with perinatal asphyxia of whom, 800,000 die, and the same number experience adverse clinical outcomes (4). The mortality and morbidity rates among patients with moderate or severe Hypoxic Ischemic Encephalopathy (HIE) is very high. Half of patients with severe HIE will die, while almost all survivors suffer from neuro-developmental deficits, cerebral palsy, epilepsy, and learning disorders (5, 6). A previous two-yr follow-up study has reported 26% mortality in asphyxic neonates, with 28% experiencing developmental delay (3). Several pathophysiologic mechanisms of brain damage in neonates are linked to HIE. Early assessment of the severity of a HIE-induced acute brain injury can be very useful for the prevention or treatment decisions in such neonates (7). The prediction of perinatal asphyxia is done using multiple assessments including the electronic monitoring of fetal heart rate during labor, cord or fetus pH measurements, meconium-stained amniotic fluid, Apgar score, the severity of HIE, prooxidant Antioxidant Balance (PAB), blood markers (Nucleated red blood cells (NRBC) in umbilical cord blood), and multiple organs impairments. None of these factors alone are sufficient and combinations of parameters are clinically used for early diagnosis of perinatal asphyxia (8, 9). Recent studies have focused on the inflammatory cytokines such as IL-1β, IL6, and IL8 for early diagnosis of brain damage. As interleukins (ILs) are known as one of the early inflammatory responses to infections, they are potentially important in early diagnosis and hence proper management of both infectious and non-infectious conditions before the establishment of fulminant stage. The inflammatory cytokines are involved in the biochemical pathways leading to ischemic-hypoxic injury (10, 11). The role of inflammation in neonatal Central Nervous System (CNS) injuries as well as the role of cytokines as mediators of injuries have been identified (12). It is likely that the pathophysiology of perinatal asphyxia has a close relationship with the inflammatory mediators such as cytokines (13). Many of these cytokines such as IL-1β, IL6, IL8, IL10, and IL12 increase during the inflammatory responses (14). A study has shown that cytokines cause brain damage through direct injury to the white matter, weakening the germinal matrix endothelium, brain hemorrhage, and inflammatory reactions caused by microglia and astrocytes (15). Although cytokines play a role in the regulation of cell apoptosis in CNS as well as leukocyte differentiation, proliferation, and infiltration, the precise role of pro-inflammatory cytokines such as IL6 as the main mediator in the development of brain damage is still unknown (16). Although several studies have been conducted on the relationship between the ILs and infectious conditions, however, comparison of their value in confirmation or rule out of the diagnosis of infection has not been fully discussed yet. Identification of biochemical markers such as IL may be useful for early diagnosis of asphyxia. Early diagnosis of perinatal asphyxia helps to provide better health care and improved outcomes. Brain injury is also a common cause of sever morbidity with poor outcomes and high mortality during the perinatal period.

The current systematic review was conducted aiming at the identification of neonatal perinatal asphyxia by IL levels.

## 2. Materials and Methods

### Selection of ILs for the diagnosis of neonatal asphyxia

After an initial review of the literature, a list of ILs was prepared to conduct a systematic review. The articles examining the role of ILs in the diagnosis of neonatal asphyxia were studied. In this regard, articles containing ILs such as IL-1, IL-6, or combinations of both were analyzed for the diagnosis of neonatal asphyxia.

### Search strategy

PubMed, Cochrane Library, Web of Science, Embase, and Scopus databases were used for this systematic review. “Asphyxia,” “neonatal asphyxia,” “perinatal asphyxia,” “interleukin,” and “diagnosis” were the search keywords. All articles in English and Persian between and consisting 1997 and 2016 were searched.

### Inclusion criteria

Articles with the following criteria were selected for this review: (1) Neonates as the study population; (2) Neonatal asphyxia as the specified study; (3) ILs must be examined for the diagnosis or prediction of neonatal asphyxia in umbilical cord blood, serum, or Cerebro Spinal Fluid (CSF); and (4) Adequate information from the test results.

### Exclusion criteria

Animal studies; articles that used other markers for the diagnosis of asphyxia rather than ILs; and articles with only abstract available were excluded from the study.

### Data extraction and quality assessment of the articles

**Figure 1:**
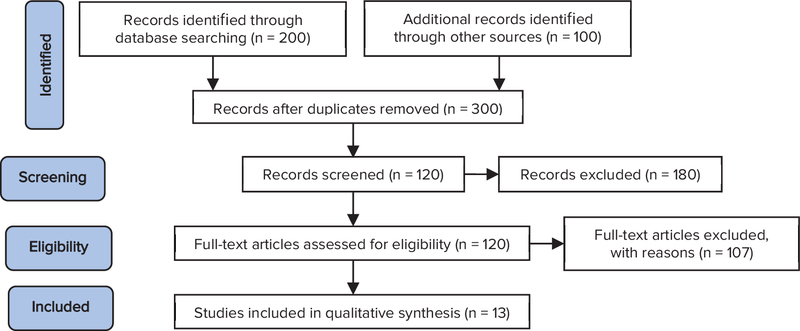
Search strategy and selected articles.

The full-text articles were downloaded and the extracted data were collected in Microsoft Excel with the following titles: the authors' names and surnames, years of publication, methods, study areas, case groups, control groups, type of IL, locations of sampling, time for measuring IL, sensitivity, specificity; positive predictive value, negative predictive value, and the results of the investigation. Initially, 300 studies were collected using EndNote software and the duplicate articles (n = 115) were excluded from the review. According to the title and abstract, 65 more articles were excluded. A total of 107 other articles were also removed due to incomplete data, unavailability of full text, animal studies, uncertainty of the study type and the target group. Finally, 13 related articles underwent further analysis (Figure 1).

## 3. Results

A total of 13 out of 300 articles with a total sample size of 1120 neonates were examined. Eight articles discussed IL6 (61.54%), two studies (15.38%) described about IL-1β, and three articles (23.08%) were related to the combination of both ILs.

### The number of the studies on ILs

The review of studies conducted from 1997 to 2016 showed that IL6 is the most frequently studied biomarker among IL family. Interleukins had been measured in 10 studies on serum samples (76.92%), 2 on CSF samples (15.39%), and 1 on umbilical cord blood samples (7.69%). All studies were prospective ones.

### Heterogeneity of the articles

**Figure 2:**
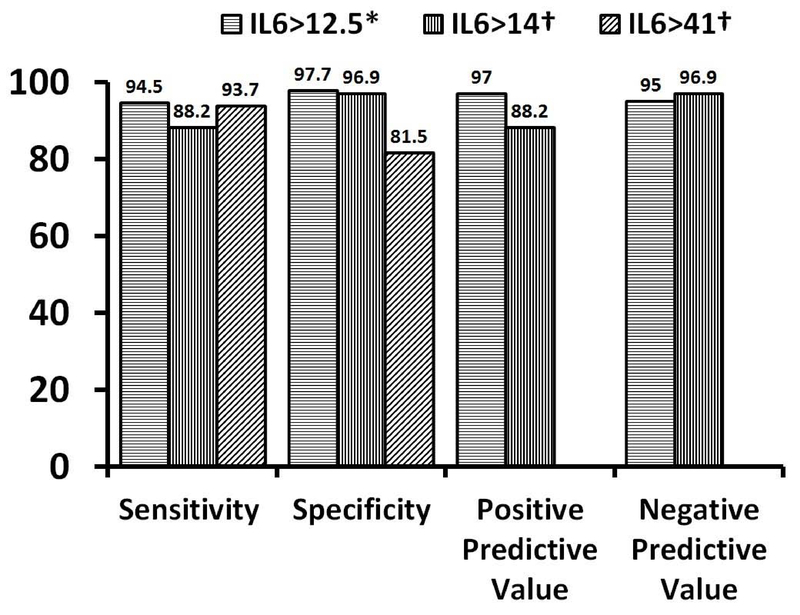
The sensitivity, specificity, positive, and negative predictive values (%) of IL6 in prediction of asphyxia${}^{*.}$ and its adverse outcomes${}^{†}$.

Studies on the relationship between IL and diagnosis of perinatal asphyxia were different in terms of inclusion criteria, sample size, sampling location, time of assessment, and the diagnostic value of ILs. Only five studies have discussed the diagnostic value of IL (38.46%). The cutoff values for both IL6 and IL-1β were 11.91-100, and negative predictive values of ILs were different in the articles under study (Figure 2, Table I).

### IL6 (eight studies)

In a study on serum levels of IL6 at birth, 24 and 48 hr after delivery for 37 neonates with perinatal asphyxia and 45 healthy neonates serum concentrations of IL6 was reported as 43 times higher in asphyxiated neonates with HIE and 1.9 times higher in asphyxiated neonates without HIE compared to the healthy neonates. Serum concentrations of IL6 were increased in neonates with asphyxia after birth and this was associated with the severity of encephalopathy and poor outcomes. The sensitivity, specificity, positive, and negative predictive value of IL6 in prediction of asphyxia and its adverse outcomes are shown in Figure 1. So following cerebrospinal system injury, IL6 plays an important role and its serum concentrations can be a useful predictor of HIE outcomes (1). Another study comparing the IL6 in 50 neonates with non-infectious perinatal asphyxia with 113 healthy neonates at similar time points showed that the average concentrations of IL6 in neonates with HIE were 376 times higher than the healthy babies and 5.5 times higher in neonates with asphyxia without HIE. A critical relationship was also found between IL6 and the degree of HIE as well as neuro-developmental outcomes at two yr. Regardless of the outcomes, serum IL6 concentration was significantly lower in the first 24 and 48 hr after birth. Umbilical cord concentration of IL6 > 100 pg/ml had the sensitivity and specificity of 70.8% and 80.9%, respectively, in predicting moderate to severe HIE. It was concluded that IL6 measurement in the umbilical cord blood of newborns with perinatal asphyxia may be useful in early detection of neonates with high risk of brain damage and adverse outcomes (17). A study on the neurological markers (IL6) in perinatal asphyxia and its relationship with different stages of HIE was performed on 100 asphyxic and 100 healthy neonates with blood samplings in the first and third days of birth. The results showed that the average concentration of serum IL6 on the third day of birth of asphyxic neonates declined. There was also a negative relationship between IL6 concentrations in the first and third days of birth. The IL6 concentration had a reverse correlation with the HIE stages (Stages 1-3) within the 1st to the 3rd day of birth. In addition, there was a negative relationship between the first and third day of life in terms of IL6 concentrations in various stages of HIE. It was concluded that IL6 concentration increases after birth asphyxia, and this increase is associated with the severity of HIE and poor outcomes (18). In a study on the predictors of early HIE in Egypt, serum IL6 was measured in the first 12 hr of life in 27 perinatal asphyxic as well as 25 healthy neonates.

 The HIE group showed significantly higher IL6 levels compared to the controls. IL6 measurements have also been performed in the CSF of full-term neonates with HIE. This prospective study was performed on 20 healthy newborns (no sepsis or meningitis; 1 and 2 min Apgar scores ≥ 9) and 15 neonates in the case group (with asphyxia and Apgar score of ≤ 4 in the first minute and ≤ 6 in the fifth minute, umbilical cord blood pH < 7.20 or lactate > 3 mmol/liter in the umbilical artery blood and need for artificial ventilation for at least two minutes after birth). The CSF samples were collected within the first 48 hr after birth to identify IL6. The results showed that the average IL6 in the case group (157.5 pg/ml) was significantly higher than the control group (4.1 pg/ml). The researchers concluded that IL6 levels in term neonates with HIE is higher than the control group (19). A different study aiming at the evaluation of IL6 in CSF after perinatal asphyxia and its relationship with early and late nervous observations was conducted on 20 infants, among whom, 3 cases had no HIE (stage 0), 5 infants were at stage 1, 6 infants at stage 2, and 6 infants at stage 3 of HIE. The IL6 concentration in CSF (from 8 to 90 hr of birth) in infants at stage 3 of HIE was higher than those at stage 0 to 2. Infants with brain damage and adverse outcomes showed higher CSF levels of IL6. The increase in CSF IL6 after perinatal asphyxia was related to the intensity of HIE, brain damage, and neurological outcomes. The researchers concluded that IL6 may be involved in hypoxic-ischemic brain damage (20). A study on the clinical significance of serum IL6 in neonates with HIE was performed on 74 neonates with HIE along with 74 healthy neonates. An increase in the inflammatory mediators was associated with the severity of the disease and positively correlated with prognosis. They reported high levels of IL6 in neonates with HIE. High concentrations of IL6 in infants with HIE suggests that these inflammatory mediators play an important role in the development and prognosis of the disease (21). Serum concentrations of 50 asphyxic and 20 healthy neonates were measured on days 1, 3, and 7 after birth in a study on the relationship between IL6 and brain damage in perinatal asphyxia. The results showed that IL6 levels in asphyxic neonates reduced within a week after birth and reverted to the normal level on day 7 after birth; however, the IL6 levels were significantly lower in neonates with brain damage compared with neonates with no brain injury. As a conclusion, IL6 levels increase in neonatal asphyxia; hence, it can be involved in the pathophysiology of neonatal asphyxia (22).

### IL-1β  (two articles)

**Figure 3:**
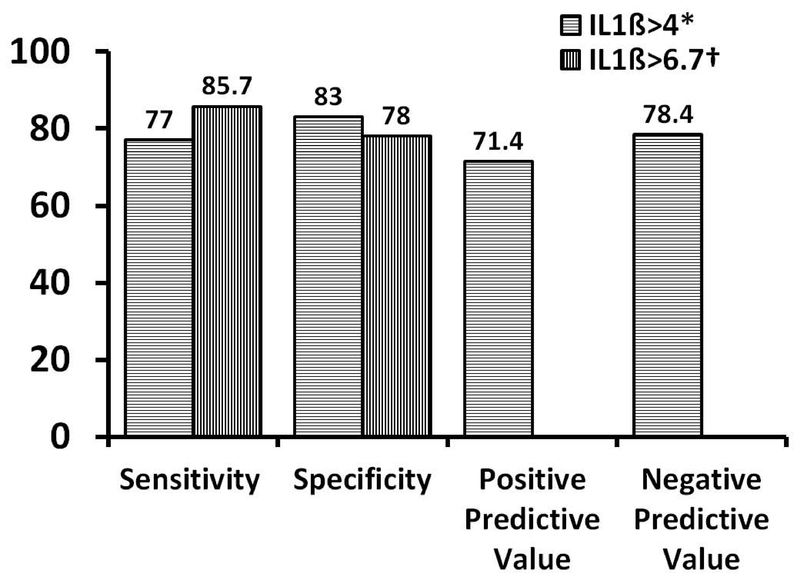
The sensitivity, specificity, positive, and negative predictive values (%) of IL-1β in the prediction of the occurrence of asphyxia${}^{*.}$ and its adverse outcomes${}^{†}$.

Serum levels of IL-1β were studied in 38 neonates with non-infectious perinatal asphyxia (blood pH < 7.2, low Apgar score, and fetal distress symptoms) and 41 healthy neonates (natural babies with no clinical signs of asphyxia during the first week after delivery) at birth as well as 24 and 48 hr after the delivery. The serum levels of IL-1β in neonates with HIE were five times more than asphyxiated newborns without HIE and six times higher than healthy babies. Also, a significant relationship was found between IL-1β and neonatal outcomes at discharge. The sensitivity, specificity, positive, and negative predictive value of IL-1β in prediction of the occurrence of asphyxia and its adverse outcomes are shown in Figure 3.

**Table 1:** Summary of the studies conducted on the diagnosis of perinatal asphyxia by assessing interleukins (ILs)


**Quadas score**	**Results**	**Negative predictive value**	**Positive predictive value**	**Specificity**	**Sensitivity**	**Turning point**	**Time of Assessment**	**Markers**	**Control Group**	**Case Group**	**Place**	**Author/ Year**
13	Simultaneous evaluation of IL-1β and IL6 can improve the sensitivity of the early diagnosis of perinatal asphyxia		IL-1β: 1.89%; IL6: 6.81%	IL-1β: 71%; IL66.5. 80%	IL-1β: 3.35%; IL6: 6.5.11.91%	At birth	Serum UL-1β and IL 6	47 healthy infants	38 Uninfected infants with perinatal asphyxia	Iran	Boskabadi *et al*. (2016)
12	IL6 serum is useful predictors for the outcomes of HIE and intensity of perinatal asphyxia.	95%	97%	97.70%	94.50%	IL6: 12.5pg/ml	At birth, 24 and 48 hr after delivery	IL 6 serum	45 healthy infants	37 uninfected infants with perinatal asphyxia	Iran	Boskabadi *et al*. (2010)
		81.50%	93.70%	IL6: 41pg/ml			
	96.90%	88.20%	96.90%	88.20%	IL6: 41pg/ml + of moderate or severe asphyxia			
13	IL-1β serum is predictors of term neurological consequences and intensity of perinatal asphyxia.	4.78%	4.71%	38%	77%	14 pg/ml	At birth, 24 and 48 hr after delivery	IL 6 serum	41 healthy infants	38 uninfected infants with perinatal asphyxia	Iran	Boskabadi *et al*. (2010)
		78%	7.85%	7.6 pg/ml			
11	IL6 assessment in umbilical cord blood is useful for early diagnosis of infants at high risk of brain damage and adverse consequences.		80.90%	70.80%	100 pg/ml	At birth, 24 and 48 hr after delivery	IL 6 serum	113 healthy infants	50 uninfected infants with perinatal asphyxia	Italy	Chiesa *et al*. (2003)
12	IL6 is increased after asphyxia at birth which is associated with the severity of HIE and poor outcomes.			The first and third days of life	IL 6 serum	100 healthy infants	50 infants with perinatal asphyxia	India	Paliwal *et al*. (2014)
11	IL6 and TNF-α levels are more in term infants or HIE than with that in healthy infants.			48 hr first birth	IL6 in CSF	20 healthy infants	15 infants with HIE	Brazil	Silveira *et al*. (2003)
13	IL-1β is associated with central nervous system (CNS) damage after hypoxia and can be a useful predictor for HIE.				IL-1β in plasma and CSF	11 healthy infants	19 infants (14 infants with abnormal development and 5 infant deaths)	Turkey	Oygür *et al*. (1998)
12	The assessment of IL6 level may be useful in early diagnosis of perinatal asphyxia			12 hr first birth	IL6 serum	25 healthy infants	27 infants with evidence of HIE	Egypt	El Farargy *et al*. (2014)
11	High levels of IL6, TNF and CRP was observed in neonates with HIE.				IL6 serum	74 healthy infants	74 infants with HIE	China	Shang *et al*.(2014)
13	Increased levels of pro-inflammatory cytokines in neonates with asphyxia is indicative of future neurological disorders			24 hr first, third and seventh days of birth	UL-1β and IL 6 Serum	28 healthy infants	29 infants with perinatal asphyxia	Greece	Skouteli *et al*. (2001)
12	An increases in IL6 in CSF after perinatal asphyxia is associated with severe HIE, brain damage, and neurological outcome.			8 and 90 hr after delivery	IL 6 serum	14 healthy infants	5 infants with brain injury symptoms	Spain	Martin- Ancel *et al*. (1997)
13	IL6 level increases in neonatal asphyxia.			1${}^{\mathrm{st}}$, 3${}^{\mathrm{rd}}$ and 7th days after birth	IL 6 serum	20 healthy infants	50 infants with perinatal asphyxia	China	YH Zhou *et al*. (2004)
12	Serum levels of IL1, IL6 and TNF increases in neonates with HIE.			At birth, 24 and 72 hr after delivery	IL 6 serum	55 healthy infants	29 infants with HIE	Saudi Arabia	Alsulaimani *et al*. (2015)
Note: IL-1β: Interleukin-1β; IL-6: Interleukin- 6; TNF-α: Tumor necrosis factor alpha; HIE: Hypoxic Ischemic Encephalopathy; CRP: C-reactive protein; CSF: Cerebro spinal fluid												

The researchers concluded that the increase in serum levels of IL-1β in asphyxic neonates is a predictor of poor outcomes. In other words, serum IL-1β is a predictor for the severity of perinatal asphyxia and its short-term neural outcomes (23). A different prospective study was conducted on the predictive value of plasma and CSF concentrations of IL-1β in the outcomes of 30 term neonates with HIE. Blood and CSF samples were collected within the first 24 hr after birth. Five babies died immediately after hypoxia. The neurological examination and Denver Developmental Screening Test were performed at one yr of age. Eleven neonates had normal and fourteen had abnormal neurological findings or abnormal Denver Developmental Screening Test. The results indicated that the CSF concentration of IL-1β in unhealthy infants was significantly higher than healthy ones. However, no significant difference was found in the plasma concentrations of IL-1β in two groups. Patients with CSF samples taken within six hr of hypoxia had higher levels of IL-1β compared to those with sampling after six hr of hypoxia. The researchers concluded that IL-1β level is correlated with CNS damage after hypoxia and can be a useful predictor for HIE (24).

### Combination of IL6 and IL-1β (three articles)

**Figure 4:**
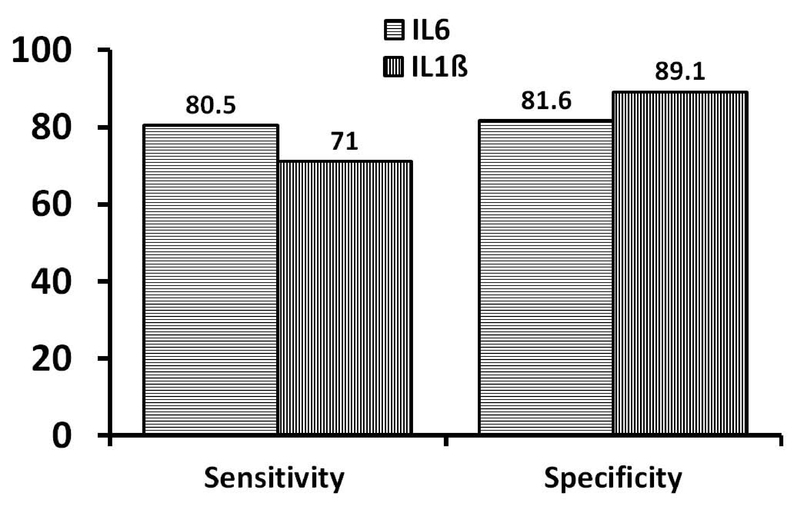
The sensitivity and specificity (%) of IL6 and IL-1β in prediction of diagnosis of perinatal asphyxia.

Combination of IL6 and IL-1β at birth was studied in 38 infectious infants with perinatal asphyxia (pH < 7.2, low Apgar score, and fetal distress symptoms) and 47 healthy infants. Serum concentrations of IL6 and IL-1β were significantly higher in infants with perinatal asphyxia compared to the healthy ones (88.15 vs. 6.74pb/ml for IL6 and 16.88 vs. 3.34pb/ml for IL-1β). The sensitivity and specificity of IL6 and IL-1β are shown in Figure 4. The turning points for IL6 and IL-1β were 11.91 pb/ml and 3.35 pb/ml, respectively. The researchers concluded that simultaneous assessment of IL6 and IL-1β can improve the sensitivity and specificity for early diagnosis of perinatal asphyxia. In addition, the assessment of the combination of IL-1β and IL6 was the best indicator for perinatal asphyxia (25).

In a different study on evaluation of the markers for early diagnosis of brain damage in the preterm low-birth-weight infants with perinatal asphyxia, 29 infants with perinatal asphyxia and 28 healthy infants were involved. Serum IL6 and IL-1β were measured in the first 24 hr and the 3rd and 7${}^{\mathrm{th}}$ days of birth. The study results showed

that serum IL6 and IL-1β were significantly higher in infants with perinatal asphyxia in the first 24 hr after birth compared to the healthy infants. Neurologic evaluation of 14 infants with perinatal asphyxia and 12 healthy ones at 18 months of age revealed that 8 infants with perinatal asphyxia had abnormal findings that were associated with serum levels of IL6 and IL-1β in 24 hr after birth. The researchers concluded that increased levels of pro-inflammatory cytokines are the primary findings for the future neurological disorders in infants with asphyxia (26). In an evaluation of the inflammatory cytokines in 55 healthy and 45 infants with HIE, serum levels of IL6 and IL-1β were measured at birth, 24, and 72 hr after birth. A significant increase was found in IL6 and IL-1β in infants with HIE at three time points (27). The combination of IL6 and IL-1β had the highest average sensitivity (92.9%) and specificity (85.43%) among diagnostic ILs in asphyxiated infants (Table II).

**Table 2:** Average diagnostic value of interleukins (IL) in relation to infant asphyxia


**Biomarker**	**Mean Cutoff (pg/ml)**	**Mean Sensitivity Limit (%)**	**Mean Specificity (%)**
IL6	>41.35	84.88	85.43
IL1-β	>4.68	77.9	83.37
IL6 ± IL1-β	>17.25	92.9	43.5

## 4. Discussion

Perinatal asphyxia is a common and serious problem that results in annually almost a million infant deaths in the world. Perinatal asphyxia may also have negative impacts on all main body organs (3). HIE increases the quick expression of inflammatory cytokines of brain (IL1 and IL6) (28). IL6 level is one of the powerful predictors of HIE outcomes such as death and long-term neuro-developmental problems (17). It appears as a considerable product among inflammatory cytokines in the pathogenesis of perinatal asphyxia (29). IL6 is as an inflammatory mediator in brain injury and plays a central role in the inflammatory responses (30). It is not clear whether IL6 has a devastating effect on neurons or healing effect after brain ischemic damage (31). IL6 may be released as a protective response after hypoxic-ischemic brain damages. It acts as a cytokine with two pro-inflammatory and anti-inflammatory properties (32). IL6 has been shown to play two roles in cerebral ischemia: as an inflammatory mediator during the acute phase and as a neurotrophic mediator within the acute and long-term stages (33). Higher levels of IL6 have been reported in infants with perinatal asphyxia and hypothermia (34). IL6 is involved in the induction of acute reactions and control the inflammatory responses causing a reduction in pro-inflammatory cytokines and an increase in anti-inflammatory molecules during acute cerebral ischemia stage (35). IL6 increases in CSF fluid in asphyxic infants and is related with the severity of HIE (20). A direct relationship has been reported between the CSF levels of IL6 and TNF-α and neurological prognosis after acute cerebral ischemia in adults (36). It has been shown that CSF levels of IL6 are significantly higher in infants with severe neurological observations compared to mild or moderate encephalopathy (20). Increased IL6 in the CSF has been shown to be related with the severity of HIE, brain injury, and neurological outcomes at 12 or 72 hr after perinatal asphyxia (20). Serum concentrations of IL6 increased approximately 12 hr after birth (1). A recent study reported an increase in serum levels of IL6 in infants with HIE (32). Animal studies have shown an increase in the peak serum levels of IL6 in rats with HIE in 6 hr after creating HIE so that the concentration of IL returns to the base level after 20 hr (28); however, researchers have not specified a certain turning point for IL6 that predicts long-term adverse outcomes. A significant relationship has been reported between the serum concentrations of IL6 and Sarnat encephalopathy grading (31). Also, increased levels of IL6, IL11, and IL13 have been reported in dried blood samples of infants with cerebral palsy (37). The results of an animal study showed that the serial injection of synthetic IL6 prevents learning disabilities and delays the loss of neurons (38). In another study, the increase in serum IL6 was associated with poor outcomes or death in infants with perinatal asphyxia (39). An increased IL6 in amniotic fluid and cord blood were associated with outcomes such as cerebral palsy and periventricular leukomalacia, respectively (40). Cohort Study of Ahearne and co-worker is the first report that measured the association of IL-16 in neonates with perinatal asphyxia at birth and long-term outcome. IL-16 is an early biomarker of severe injury that determine the long-term prognosis of infants with HIE.

Serum IL6 had 86% positive predictive value and 100% specificity in predicting moderate to severe HIE (41). Available studies have indicated that IL6> 41 pg/dL had sensitivity and specificity of 88.84% and 85.43%, respectively, in the diagnosis of neonatal asphyxia. Therefore, the highest sensitivity and specificity are for the diagnosis of asphyxia related to IL6. IL-1β in umbilical cord is a major bio-outcome for brain injury whose levels are significantly high in infants with HIE and predict severe HIE and adverse outcomes in 6 to 12 months of age (42).

Increased CSF concentrations of IL-1β are associated with neurologic outcomes after perinatal asphyxia. It could be concluded that IL-1β has neurotrophic and neuroprotective results. However, it is not clear whether IL-1β is involved in the destruction or repair of neurons after ischemic brain injuries (31). CSF levels of TNF-α and IL-1β were higher in term infants with HIE who developed nervous disorders in one age (43). Neuroprotective antagonist effects of IL-1β receptor against brain damages before or after exposure to hypoxia have already been reported (44). The available studies showed that IL-1β> 4.7 pg/dl had sensitivity and specificity of 78% and 83%, respectively, in the diagnosis of neonatal asphyxia. IL-1β and IL6 are significantly increased in birth asphyxia and the rate of increase is associated with the severity of encephalopathy. Simultaneous assessment of IL-1β and IL6 is the best indicator of perinatal asphyxia (25). IL-1β and IL6 levels have been reported to be significantly higher in infants with perinatal asphyxia compared to the control group in 24 hr after birth (26). In another study, serum levels of IL6, IL8, and IL10 were higher in infants with severe asphyxia (death or poor outcome) than those with asphyxia but no poor outcome (39). Serum concentrations of IL-1β and IL6 were significantly higher in infants with perinatal asphyxia than that in healthy infants. Simultaneous assessment of IL-1β and IL6 improved the sensitivity and specificity of early diagnosis of perinatal asphyxia. Assessment of combined IL-1β and IL6 was suggested as the best indicator of perinatal asphyxia (25). Available studies indicated that simultaneous assessment of IL-1β and IL6 had the sensitivity of 93% and specificity of 43.5% in the diagnosis of neonatal asphyxia. The current review is the only study that has examined the role of IL in the diagnosis of perinatal asphyxia. Limitations of this study include the lack of access to unpublished articles and reports, inappropriate and low-quality reports, limited number of articles and infeasibility of accurate judgments about their effectiveness, lack of similar definitions for case groups, lack of cutoff point calculations, and lack of diagnostic value of IL in all the studies under review.

## 5. Conclusion

Serum and CSF concentrations of interleukins IL6 and IL-1β increased after asphyxia and the rate of increase was associated with the severity of asphyxia and adverse outcomes. Therefore, combination of IL6 and IL-1β can be used as a potential substantially powerful marker for early diagnosis of perinatal asphyxia. Further studies are required in order to identify more involved ILs and standardize their cutoff values in early diagnosis of neonatal asphyxia.

##  Conflict of Interest

The authors declare no conflict of interest.
